# Aggregate blood pressure responses to serial dietary sodium and potassium intervention: defining responses using independent component analysis

**DOI:** 10.1186/s12863-015-0226-8

**Published:** 2015-06-20

**Authors:** Gengsheng Chen, Lisa de las Fuentes, Chi C. Gu, Jiang He, Dongfeng Gu, Tanika Kelly, James Hixson, Cashell Jacquish, D. C. Rao, Treva K. Rice

**Affiliations:** Washington University in St. Louis School of Medicine, St. Louis, MO USA; Tulane University Health Sciences Center, New Orleans, LA USA; Chinese Academy of Medical Sciences, Beijing, China; University of Texas Health Sciences Center at Houston, Houston, TX USA; National Heart, Lung, and Blood Institute, Bethesda, MD USA

**Keywords:** Blood pressure, Cardiovascular diseases, Hypertension, Independent component analysis

## Abstract

**Background:**

Hypertension is a complex trait that often co-occurs with other conditions such as obesity and is affected by genetic and environmental factors. Aggregate indices such as principal components among these variables and their responses to environmental interventions may represent novel information that is potentially useful for genetic studies.

**Results:**

In this study of families participating in the Genetic Epidemiology Network of Salt Sensitivity (GenSalt) Study, blood pressure (BP) responses to dietary sodium interventions are explored. Independent component analysis (ICA) was applied to 20 variables indexing obesity and BP measured at baseline and during low sodium, high sodium and high sodium plus potassium dietary intervention periods. A “heat map” protocol that classifies subjects based on risk for hypertension is used to interpret the extracted components. ICA and heat map suggest four components best describe the data: (1) systolic hypertension, (2) general hypertension, (3) response to sodium intervention and (4) obesity. The largest heritabilities are for the systolic (64 %) and general hypertension (56 %) components. There is a pattern of higher heritability for the component response to intervention (40–42 %) as compared to those for the traditional intervention responses computed as delta scores (24 %–40 %).

**Conclusions:**

In summary, the present study provides intermediate phenotypes that are heritable. Using these derived components may prove useful in gene discovery applications.

**Electronic supplementary material:**

The online version of this article (doi:10.1186/s12863-015-0226-8) contains supplementary material, which is available to authorized users.

## Background

Hypertension is a major risk factor for many cardiovascular diseases. [1, 2] Moreover, there is evidence for substantial heritability as well as environmental causes underlying these diseases. [3–5] For example, an environmental intervention of reduced sodium and/or increased potassium intake can lead to reduced systolic and diastolic blood pressure (BP) [6–11], and familial factors in part underlie these BP responses to sodium intervention. [11–16] Moreover, significant inter-correlations among this set of variables suggest a complex underlying network involving multiple genes, multiple environments and gene-by-environment interactions [17]. However, the structure and mechanisms underlying such complex networks are unclear. Recent studies investigating these issues focus on endophenotypes, defined here as intermediate traits that are derived from observed measures [18, 19]. Such endophenotypes may provide clues to the mechanisms and genetic architecture underlying clinical diseases, and factor analysis is a tool that may be used to extract these hidden components.

Traditionally, principal component analysis (PCA) [20] is used to identify uncorrelated factors. PCA assumes mutivariate normality among the variables. However, if multivariate normality is violated the resulting components may not be independent and thus not uniquely interpretable.  The related method of independent component analysis (ICA) [21] produces uncorrelated and independent component, even when multivariate normality is violated [22, 23]. Consequently, in the investigation reported here, ICA is used to construct factors that may serve as endophenotypes to help identify the underlying genetic architecture.

The purpose of this report is to construct endophenotypes using ICA analysis of several obesity and BP measures during a sodium intervention test in the GenSalt study, and assess their utility using heritability analysis. GenSalt is a dietary sodium/potassium intervention study on blood pressure levels in rural Chinese families [24]. After baseline, there were 3 intervention periods (low and high sodium and a potassium supplement). ICA analysis extracted 4 endophenotypes (component factors) which were clinically interpreted using a heat map protocol. The utilities of the endophenotypes for future genetic applications were evaluated by heritability analysis.

## Methods

### Study subjects

The GenSalt study was designed to identify genes related to variation in BP responses to dietary sodium [24]. The study was conducted in six rural Chinese provinces around Beijing using probands (i.e. high BP) and their family members. Eligible probands (see [24]) were 18–60 years of age, had high BP (SBP ≥ 130 mm Hg and/or DBP ≥ 85 mm Hg), and had at least 1 parent and at least 1 sibling who could participate, although additional family members (spouses and offspring) also could participate. Eligible siblings, spouses and offspring were aged 18–60 years (≥16 for offspring) and lived in the same village with the proband. Exclusion criteria for all participants are listed elsewhere [24] but generally exclude those with stage 2 hypertension or on antihypertensive medications, having a history of cardiovascular disease, diabetes, kidney disease, or liver disease, who were pregnant or heavy alcohol drinkers, or on a low-sodium diet. Only probands and their siblings, spouses and offspring (i.e. not parents) participated in the dietary intervention. A total of 3,150 individuals from 658 families were included in the study at baseline, but only 1,906 individuals participated in the dietary sodium intervention. The reduced sample size (1,906 versus 3,150) primarily is due to the parents of the probands (older generation) not participating in the intervention by design. The rational for this study design (omitting probands parents from the intervention) was that the siblings of the probands would provide the most useful information [24]. Isolated missing data occurred for 64 individuals, resulting in 1,842 individuals with complete data available for analyses.

This study protocol was approved by the Institutional Review Boards at all participating institutions, including Washington University in St. Louis and Tulane University, and all participants gave their informed consent.

### Dietary sodium intervention

In the first 3-day baseline observation phase participants consumed their usual diet. Then, participants received a low sodium diet (51.3 mmol of sodium per day) for 1 week, followed by a high sodium diet (307.8 mmol of sodium per day) for 1 week, and finally a high sodium diet plus a potassium supplement (60 mmol potassium) for 1 week, see [24] for further details about the intervention. BP was measured (see below) during baseline (B) and during each of the low sodium (L), high sodium (H), and potassium supplement (K) intervention periods.

### Phenotypes

A variety of demographic, pedigree, medical history, lifestyle risk factor, anthropometric, blood and urine data was collected on all study participants. For the current analysis, a total of 20 phenotypes (plus age) are used (see Table 1). There are 2 anthropomorphic measures (body mass index (BMI) and waist circumference (WST]) and 12 BP measures for systolic (SBP), diastolic (DBP) and pulse pressure (PP = SBP-DBP). BP was measured by trained and certified observers according to a common protocol adapted from the American Heart Association [25]. Participants were seated and BP was measured after a 5-min rest using a random-zero sphygmomanometer with an appropriate cuff size. Participants were advised to avoid alcohol, cigarettes, coffee/tea and exercise for at least 30 min prior to their BP assessment. At baseline (B), BP was measured during the morning and averaged across the 3 baseline days (B_SBP, B_DBP, and B_PP). During each of the intervention weeks, BP was measured on the mornings of days 2, 5, 6, and 7. However, the analysis variable was the average across only days 5, 6, and 7 (L_SBP, L_DBP and L_PP for low-sodium week, H_SBP, H_DBP and H_PP for high sodium week, and K_SBP, K_DBP and K_PP for potassium supplement week).

**Table 1:** Variable characteristics

	Males (*N* = 974)			Females (*N* = 868)		
Variable	Mean	SD	SE	Mean	SD	SE
Demographic						
AGE	39.2	9.5	0.3	38.2	9.3	0.3
Anthropometric						
B2_WST	81.9	9.9	0.3	78.5	9.5	0.3
B_BMI	23.2	3.1	0.1	23.5	3.2	0.1
Hemodynamic						
B_SBP	118.6	12.5	0.4	115.0	15.5	0.5
B_DBP	75.6	9.8	0.3	71.8	10.5	0.3
L_SBP	113.3	11.1	0.4	109.3	13.1	0.4
L_DBP	73.2	9.3	0.3	68.6	9.6	0.3
H_SBP	117.8	12.1	0.4	114.5	15.0	0.5
H_DBP	74.5	9.9	0.3	71.1	10.3	0.4
K_SBP	114.1	11.6	0.4	111.1	14.3	0.5
K_DBP	73.4	9.5	0.3	69.4	9.9	0.3
B_PP	43.0	9.0	0.3	43.2^a^	9.8	0.3
L_PP	40.1	8.9	0.3	40.7^a^	9.4	0.3
H_PP	43.2	8.8	0.3	43.4^a^	9.5	0.3
K_PP	40.8	8.9	0.3	41.7	9.6	0.3
D_LH_SBP	4.4	5.9	0.2	5.3	6.1	0.2
D_LH_DBP	1.3	5.5	0.2	2.5	5.3	0.2
D_HK_SBP	−3.6	5.2	0.2	−3.4	5.8	0.2
D_HK_DBP	−1.2	4.7	0.2	−1.7	4.4	0.2
D_BL_SBP	−5.3	6.9	0.2	−5.7^a^	7.1	0.2
D_BL_DBP	−2.4	5.7	0.2	−3.2	5.3	0.2

Abbreviations: SD = standard deviation; SE = standard error; AGE = age in years; SBP = Systolic Blood Pressure (mm Hg); DBP = Diastolic Blood Pressure (mm Hg); PP = Pulse Pressure = SBP-DBP; BMI = Body Mass Index (kg/m^2^); WST = waist circumference (cm); B_ = Baseline; L_ = Low sodium intervention; H_ = High sodium intervention; K_ = Potassium supplement intervention; D_BL_ = response (delta), Low minus Baseline; D_LH_ = response (delta), High minus Low sodium; D_HK_ = response (delta), Potassium minus High sodium

^a^All mean values are significantly different across genders at 0.05 except as noted by asterisk using standard error comparison

### Statistical analysis

#### Age adjustments and distributional properties

The anthropometric and hemodynamic variables are adjusted for a polynomial in age (age, age^2^, age^3^) separately by gender groups after exploring distributional properties. First, sparse outliers (see definition in supplemental material) were temporarily set aside. A given phenotype was regressed on a polynomial in age retaining only significant terms. Age effects in the residual variance (heteroscedasticity) were also examined. A given analysis phenotype was the standardized residual from this regression. Skewness and kurtosis of these standardized variables was also explored to ensure each variable was normally distributed.

#### ICA and PCA

Independent components analysis (ICA) is used to identify independent components using correlated variables. The 20 correlated variables include: SBP, DBP and PP, each measured at baseline and during each of the 3 intervention periods (*N* = 12 BP variables), BMI and waist circumference (*N* = 2 variables), and the SBP and DBP responses to adjacent intervention weeks (i.e. baseline to low, low to high, and high to potassium supplement, *N* = 6). The software R (fastICA) [21] is used to extract the independent components.

The optimal number of components will be determined using the screen criteria. A preliminary principal components analysis (PCA, using SAS Proc Factor) [20, 26] is performed for the purpose of generating the scree plot [27] (see supplemental material). The scree method plots the relationship between the eigenvalues and the number of factors. The optimal number of factors is where the line stops its precipitous descent and levels off. Each ICA is subscripted by a first digit to denote the predefined number of components (2, 3 or 4), and a second digit to label the individual component (i.e. ICA31 represents the first component of a 3-component solution).

#### Identification of component hypertension traits

Several methods are used to define the component traits. First, loadings of each component on the original phenotypes are used to identify enriched representation of any particular phenotypic groups. Second, clinical characteristics of each component are classified using a heat map (Excel, Microsoft Office, version 2010, Redmond, WA). The heat map depicts the means of the original 20 phenotypes separately for two groups, with high risk values shown in red and low risk values in green. The brightness of the colors is determined by within-variable comparisons across groups where more intense colors represent the extremes of the variable range.

#### Analysis of heritability

Heritability of the adjusted and standardized variables is computed using maximum likelihood methods under a variance components model as parameterized in the computer program QTDT [28]. In this model the residual phenotypic variance is stratified into an additive familial effect (heritability) and the remaining non-familial effects. Heritability is defined as the percentage of the total variance that is due to familial factors. QTDT allows for complex family structures (i.e. extended pedigrees). GenSalt family structures ranged from 2- to 3- generations with the total number of individuals in each pedigree ranging from 1 through 22. Covariates in the heritability model include gender, generation, field center and average room temperature (i.e. these effects were partitioned out prior to computing heritability).

## Results

The descriptive statistics for the variables are presented in Table 1 (separately by males and females) and in Additional file 1: Table S2 of the supplemental materials. As shown, there are 974 males and 868 females. In general, females have a larger BMI, are younger and have lower BP levels than males. Consequently, age adjustments are performed separately for males and females. Age is a significant covariate for most of the variables (see Additional file 1: Table S1 in supplemental materials) accounting for up to 19 % of the phenotypic variation. Also, the mean BP responses for probands are larger than those for the other groups (spouses, siblings and offspring), as expected since probands were selected for high blood pressure. Additionally, there were few deviations from normality based on the skewness and kurtosis of the individual analysis variables (see Additional file 1: Table S4 in supplemental material).

### Factor interpretation

**Fig. 1:**
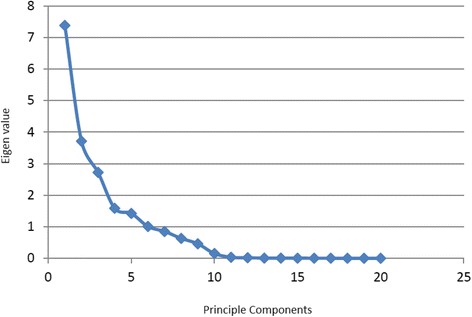
Scree plot of Eigen values. Although there are 6 eigenvalues greater than 1.0, the graphical Scree plot indicates 4 components may best represent the data

**Fig. 2:**
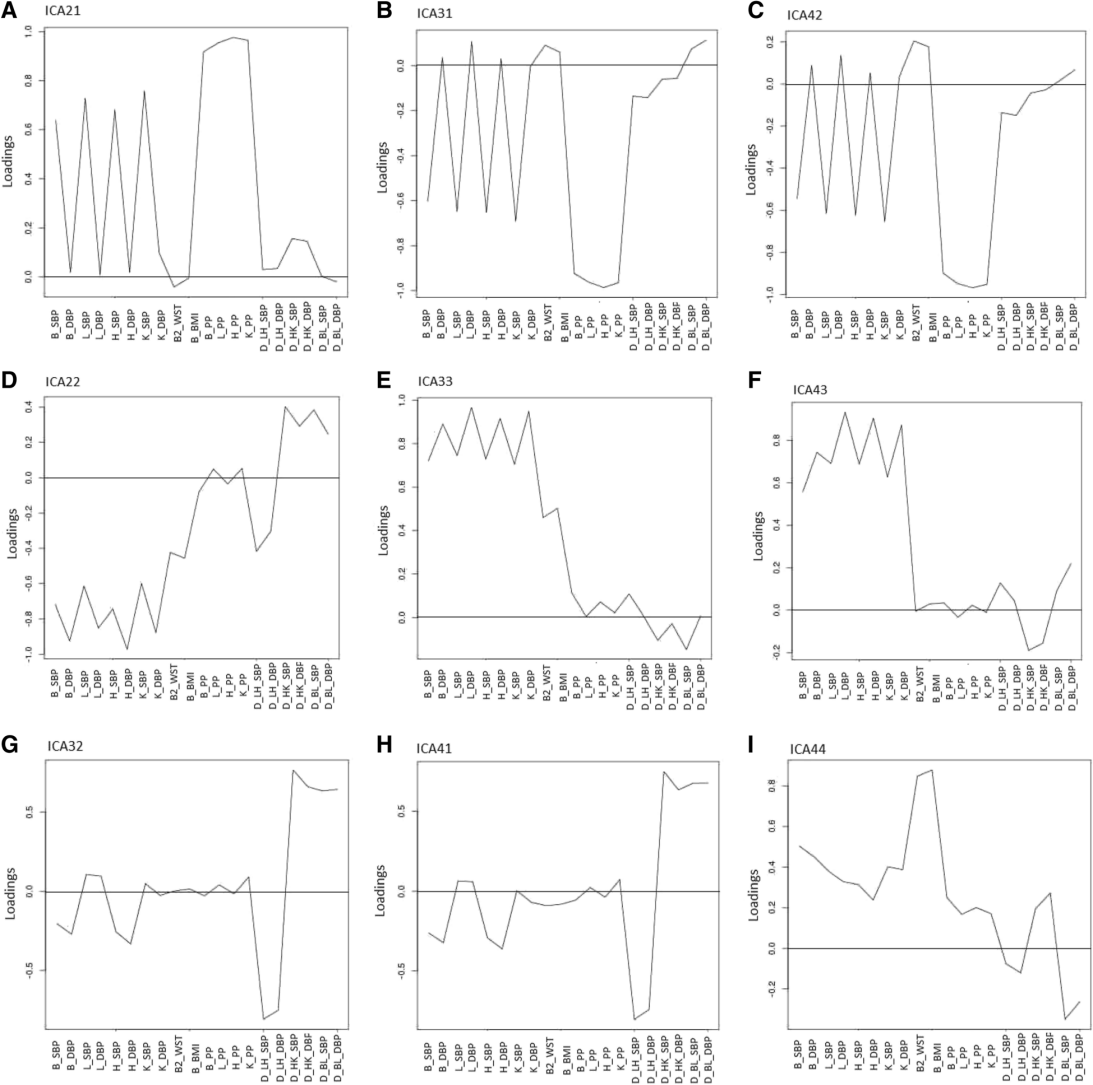
Graphic loadings for the 2-, 3- and 4-component solutions for ICA. The top row (panels **a**, **b**, and **c**) represent solutions for the "Systolic Hypertension" component (A = ICA21 = 1st factor of the 2-factor solution, B = ICA31 = 1st factor of the 3-factor solution, and C = ICA42 = 2nd factor of the 4-factor solution).  Similarly, the middle row (panels **d**, **e**, and **f**) represent solutions for the "Generalized Hypertension component (D = 2nd factor of the 2-factor solution, E = 3rd factor of the 3-factor solution, and F = 3rd factor of the 4-factor solution).  The first two panels of the bottom row (**g** and **h**) represent the "Response to Intervention" component (G = 2nd factor of the 3-factor solution and H = 1st factor of the 4-factor solution).  Finally, panel **i** represented the "Adiposity" component (4th factor of the 4-factor solution)

**Fig. 3:**
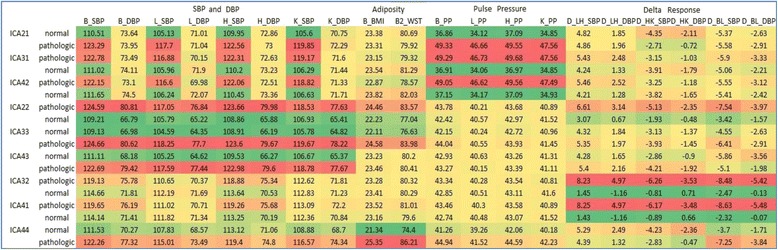
Heat map for 2-, 3- and 4-component solutions. These maps are grouped by pattern for ease of interpretation. For each extracted component, the cohort was sorted by ICA scores and split into two equal subsamples by the median; thus, the two bars for a given component represents the lowest and highest ICA scores, labeled low risk and high risk in the figure. For each median group, the average scores for each of the 20 variables are provided. High risk means for the individual variables (e.g. higher BP values) are depicted as red and low risk means are shown in green. Brightness of color denotes more extreme (high or low) scores

Table 2 shows the correlation coefficients among the ICA components and Fig. 1 shows the scree plot. The scree plot clearly suggests 4 factors best represent the data (i.e. the eigen values begin to level off after 4 components). However, we present results for 2-, 3-, and 4-component solutions in order to evaluate the stability of the preferred 4-component solution. Fig. 2 depicts the graphic loadings for each of the 2-, 3-, and 4-component solutions, and Fig. 3 shows the heat map for each of the components and solutions. In Table 2, within each 2- or 3- or 4-component solution the off-diagonal elements are zero as expected since the components within each solution are derived to be independent. Informative correlations occur between solutions (e.g. between 2- and 3-components). High inter-correlations suggest that the same underlying constructs replicate across different solutions. The first pattern is among ICA21, ICA31 and ICA42; the second is among ICA22, ICA33 and ICA43; and the third is between ICA32 and ICA41. ICA44 is found only in the 4-factor solution. Thus, the “same” constructs reoccur whether there are 2-, 3- or 4-components, and suggest that the 4-component solution is stable. This “stability” is further demonstrated below where the constructs underlying the factors is discussed.

**Table 2:** Inter-correlation coefficients for different ICA components

	ICA21	ICA22	ICA31	ICA32	ICA33	ICA41	ICA42	ICA43	ICA44
ICA21	1	0	−0.980	0.141	0.102	0.120	−0.970	0.072	0.197
ICA22		1	−0.037	0.400	−0.915	0.452	−0.090	−0.773	−0.434
ICA31			1	0	0	0.014	0.990	0.010	−0.130
ICA32				1	0	0.993	0.000	0.002	0.109
ICA33					1	−0.060	0.057	0.845	0.527
ICA41						1	0	0	0
ICA42							1	0	0
ICA43								1	0
ICA44									1

#### Systolic hypertension (ICA21, ICA31, ICA42)

A comparison of the loadings for this factor (whether under the 2-, 3-, or 4-component solution, Fig. 2, top row) consistently shows more extreme loadings (either positive or negative) for all of the systolic and pulse pressure variables. Note that the positive or negative directions of the extreme loadings and the component factor values are arbitrary in ICA analysis. The heat map (Fig. 3) provides corroborating information regarding interpretation of the factor. That is, there is a strong red-green contrast between the lower and upper component factor median groups for all of the SBP and PP traits. Moreover, this pattern is consistent across the three solutions, suggesting the construct is robust regardless of the number of components extracted. Given the systolic and pulse pressure variables loading on this factor and the heat map, it is labeled a “systolic hypertension” construct.

#### Generalized hypertension (ICA22, ICA33, ICA43)

Although these inter-correlations are on average somewhat lower than those as for the first construct, the basic pattern is consistent across different solutions. The factor loadings and heat map across the 3 solutions consistently show high (positive or negative) loadings on the eight SBP and DBP measures. However, there are additional moderate contributions from other variables for two of the factors. ICA22 has additional moderate contributions from the adiposity variables and delta BP responses, ICA33 shows moderate additional contributions from the adiposity variables, and ICA43 has little additional contributions. Thus, as the number of extracted factors increases from 2 through 4, there is a pattern of increasing homogeneity among the variables. This component represents “generalized hypertension” whose precise interpretation depends on the number of factors extracted.

#### Response to sodium intervention (ICA32, ICA41)

This factor was detected only when 3 and 4 components were extracted. Both the factor loadings and the heat map suggest this factor primarily reflects the six delta response variables. It is interesting that the contrasting directions of the loadings for the response variables in this component are consistent with their effects on blood pressure. That is, the loadings for variables that tend to increase BP (LH_) are in one direction while those that tend to lower BP (BL_ and HK_) are in the other. Both factor loadings and heat map show additional moderate contributions from the high-sodium BP variables (B_ and H_). The group with the more extreme responses also has somewhat higher baseline and high-sodium BP levels. Thus, this construct represents a “response to sodium intervention”.

#### Adiposity

Finally, one factor (ICA44) was detected only when 4 components were extracted. The factor loadings (Fig. 2) and heat map (Fig. 3) suggest this factor primarily represents an “adiposity” construct. When fewer components were extracted, the adiposity variables loaded moderately on the “generalized hypertension” factor.

### Heritability

Heritabilities of the individual BP traits and ICA components are shown in Tables 3 and 4. In general, the heritabilities are higher in the intervention conditions (low sodium, high sodium and high sodium + potassium supplement) than in the baseline condition (Table 3). And, heritabilities for PP pressure tend to be higher than those for the corresponding SBP and DBP (Table 3). In general, heritabilities tend to be larger for the components as compared to the individual variables. For example, heritability for the systolic hypertension (64 %) and the generalized hypertension components (as high as 56 %) (Table 4) tend to be higher than those for SBP and DBP (23–53 %) (Table 3), although they are strictly significantly higher (based on standard error comparison) only for the delta SBP responses to low and high sodium conditions. Similarly, the heritabilities for the response to intervention component (40–42 %) tend to be higher than those for the delta variables (24–40 %). Another interesting pattern is seen for the generalized hypertension component. Here, the heritabilities increase (from 34 % to 44 % to 56 %) as the number of extracted components increases from 2 to 3 to 4.

**Table 3:** Heritabilities (+/- Standard Error) of Systolic, Diastolic and Pulse Pressure under Different Interventions

Intervention	Systolic (SBP) Heritability	Diastolic (DBP) Heritability	Pulse Pressure (PP) Heritability
Baseline (B)	0.23 (0.10)	0.25 (0.10)	0.43 (0.08)
Low Sodium (L)	0.40 (0.08)	0.50 (0.07)	0.56 (0.07)
High Sodium (H)	0.34 (0.08)	0.43 (0.08)	0.52 (0.07)
High Sodium + Potassium Supplement (K)	0.53 (0.07)	0.48 (0.07)	0.63 (0.07)

**Table 4:** Heritabilities (± Standard Error) of Responses and Component Traits

Construct	Variable	Heritability	V_e_	V_g_
Responses to Intervention (Deltas)				
Response to Low Sodium Intervention	Δ (L-B) SBP	0.26 (0.10)	0.84	0.30
	Δ(L-B) DBP	0.31 ( 0.09)	0.76	0.35
Response to High Sodium Intervention	Δ (H-L) SBP	0.24 (0.11)	0.86	0.27
	Δ (H-L) DBP	0.40 (0.08)	0.64	0.43
Response to Potassium Supplement Intervention	Δ (K-H) SBP	0.39 (0.08)	0.73	0.47
	Δ (K-H) DBP	0.34 (0.09)	0.72	0.38
Adiposity				
Body Mass Index	B_BMI	0.60 (0.07)	0.43	0.65
Waist Circumference	B_WST	0.52 (0.07)	0.51	0.55
Component Traits (ICA)				
Systolic Hypertension	ICA21	0.64 (0.06)	0.36	0.63
	ICA31	0.63 (0.06)	0.37	0.63
	ICA42	0.64 (0.06)	0.36	0.64
Generalized Hypertension + anthropometric & delta	ICA22	0.34 (0.09)	0.66	0.34
+ anthropometric	ICA33	0.44 (0.08)	0.55	0.44
	ICA43	0.56 (0.06)	0.43	0.55
Response to Intervention	ICA32	0.42 (0.07)	0.58	0.42
	ICA41	0.40 (0.08)	0.60	0.40
Adiposity	ICA44	0.49 (0.07)	0.51	0.48

Heritability (h^2^) is computed as (V_g_) / (V_e_ + V_g_), where V_e_ is environmental variance and Vg is genetic variance

## Discussion

In this study, ICA was used to explore the factor structure underlying the BP responses to a longitudinal sodium intervention study. Factors representing four basic underlying constructs were derived and were relatively stable across 2-, 3- and 4-factor solutions. Heritability analysis revealed that familial factors accounted for 40 % to 60 % of the variance in the component factors. Thus, these new endophenotypes have the potential to provide novel information about genetic architecture underlying sodium-dependent variability in hypertension, and may enhance gene discovery efforts.

ICA was chosen over PCA to extract factors since any departures from multivariate normality would result in factors that, although uncorrelated, may not be independent. This would yield factors that would be difficult to interpret clinically and less likely to represent endophenotypes that would be useful in genetic applications. The component constructs arising from this study appeared to be clinically relevant and were consistent across the alternative 2-, 3-, and 4-factor solutions. Clinical interpretation of each factor was aided by the results from the heat map which discriminated among individuals based on the means of the extracted factor value.

The first derived factor was labeled systolic hypertension and provided the strongest contrasts for SBP and PP means, across all background sodium levels. Clinically, systolic hypertension is characterized by elevated SBP (i.e. > 140), and is further classified as isolated systolic hypertension if DBP is normal (i.e. < 90) [29]. As reviewed by Kaplan [30], a larger pulse pressure reflects a greater systolic to diastolic difference and this is a major cardiovascular risk factor in the elderly caused in part by reduced contractility of major blood vessels. The component factor identified in the GenSalt study does not reach the recommended levels for a clinical definition because the probands in this sample, although having high BP, were not clinically hypertensive. However, it does distinguish among individuals with isolated higher systolic and pulse pressures. Further, the current study suggests that there is a very strong genetic component for this component factor that accounts for nearly 65 % of the total phenotypic variance.

The second construct was generalized hypertension (ICA22, ICA33 and ICA43). The heat map shows that this factor primarily discriminates among individuals based on SBP and DBP means, although additional variables also had moderate loadings depending on the number of factors extracted. For example, when only 2 factors were extracted (ICA22) there were additional moderate loadings for both adiposity and delta responses to the intervention. When 3 factors were extracted (ICA33), the delta response variables were removed from the hypertension factor and formed a third factor. Similarly, the addition of a 4th factor (ICA43) removed the adiposity variables to a new factor, leaving the hypertension factor a more homogeneous construct reflecting only SBP and DBP traits.

As reviewed by Delles et al. [31], while hypertension is influenced by genetic factors, the environment can have a powerful modulating effect which influences the magnitude of the genetic effect. This was demonstrated in the current study for the generalized hypertension factor. As the number of extracted factors increased, the number of variables that loaded on the factor decreased with an accompanying increase in the magnitude of the heritability and presumed homogeneity of the factor. The heritability for the most homogeneous solution (ICA43, 4-components) was significantly higher at 56 % than that for the most heterogeneous factor (ICA22, 2-components) at 34 %. This change was a function of both decreasing environmental variance (0.66 to 0.43) and increasing genetic variance (0.34 to 0.56, respectively) as the number of extracted factors increased. The utility of this new endophenotype as a predictor of future cardiovascular risk with a substantial genetic component should be further investigated.

The third construct is a response to the sodium intervention. This factor was stable across both the 3- and 4-factor solutions but did not appear in the 2-factor solution. The positive versus negative means in the heat map and factor loadings is consistent with the direction of effects on BP in the intervention. That is, loadings and means for the responses that lower sodium (HK_ and BL_) are opposite to those that increase sodium levels (LH_). Moreover, the group with the larger (positive or negative) responses also has somewhat higher SBP and DBP means. This suggests that individuals who are more sensitive to the sodium intervention also have higher pressures on high sodium diets, as may be expected. Genetic factors underlying this endophenotype account for over 42 % of the variance, suggesting it should be investigated to isolate the causal genes.

The final construct in this study was labeled adiposity. Both the body mass index and the waist circumference measures loaded heavily on this factor. However, the factor also includes smaller loadings for most of the BP traits and the delta responses. The heritability for this construct is 49 %, as compared to heritabilities of 60 % for BMI and 52 % for the waist circumference. As recently reviewed [32], the heritability for BMI ranges from 30–50 % in nuclear families to as high as 80 % in twin studies [33].

Heritabilities for the baseline BP traits in GenSalt (23 % to 25 %) are within range of those reported in the literature. For example, previous family studies from various ethnic backgrounds [3, 34–37], including other Chinese family studies [38] suggest between 20 % to over 50 % of the variance in SBP and DBP is due to familial factors. The estimates reported here also are very comparable to previous GenSalt reports (23 % to 32 %) in which the entire baseline sample of over 3,000 individuals were used [5] as compared to only ~1,800 individuals who completed the intervention protocol in the current report.

It is also noteworthy that the BP heritabilities, standardized for the background levels of sodium (i.e. L, H, K interventions), were nearly doubled as compared to those for the unstandardized BPs (at baseline). This pattern was strongest for SBP (increased from 23 % to as high as 53 %) and DBP (from 25 % to 48 %), but was also noted for PP (43 % to over 60 %). As previously indicated [31], the environment can have a powerful modulating effect, which can in turn influence the magnitude of the genetic effect (i.e. gene by environment interaction) as was dramatized here.

There are limitations in this study. First, while PCA analysis always extracts as the first factor the one that accounts for the most variance among the inter-correlated traits, ICA does not order the extracted components. The ICA also does not provide statistics for determining the optimal number of components to extract. However, a priori clinical knowledge about the specific disease under study can assist in making a meaningful selection of traits and in providing information about the optimal number of factors. The preliminary use of exploratory PCA also yields information about the optimal number of factors. In general, ICA may be preferred when there are uncertainties about departures from multivariate normality and to ensure appropriate clinical interpretation of independent factors.

Second, although GenSalt probands have high blood pressure, they did not meet the definition of hypertensive. Thus, some of the conclusions relating to hypertension remain to be tested in samples of hypertensive individuals. However, the fact that none of the individuals in the GenSalt were being treated for hypertension obviates other problems such as those related to the effects of medication use on blood pressure levels.

Third, in general, when using nuclear family data (i.e. parents and offspring only), the heritability estimate includes both shared genes and shared environmental (lifestyle) factors provided the latter exist. Thus, the “heritability” computed using a nuclear family design may be an overestimate of the genetic effect to the extent there are shared environments or lifestyle factors that affect BP levels. However, in the current study, our family structures are extended pedigrees consisting of up to three generations (probands and their siblings as well as the spouse and offspring of the proband). Using this extended pedigree design the heritability may be less biased as compared to a more simple design of nuclear families alone [39].

Finally, the specific results regarding the components arising from the ICA analysis may not be easily applied to other populations since our study was based on a very specific design. However, the applicability of this study is that we have found evidence of an underlying biological genetic cause for a blood pressure response to sodium. Further, this represents a “generalized” response to sodium since it includes concurrent responses to high and low sodium levels as well as to a potassium supplement. Thus, it is likely to capture genes that are common to each of these mechanisms. The identification of the genetic variants leading to this response will be investigated in follow-up studies. As reviewed in the introduction, blood pressure levels are affected by sodium intake in many populations. Whether the genetic variants identified in this Chinese sample are applicable to the general population will be investigated once the variants are identified.

## Conclusion

In summary, the present study used ICA as a robust method to extract several novel components which may enhance our ability to find the underlying genes for sodium-dependent hypertension. Certainly, as compared to the individual variables that contributed to the factors, heritabilities are higher for components indexing systolic hypertension, generalized hypertension, and BP responses to the sodium intervention. These results validate the use of these factors as novel endophenotypes in future genetic association studies.

### Availability of supporting data

The data are available from the authors upon request to and approval from the GenSalt steering committee.

## Additional file

Additional file 1:
**Additional material.** 1. Optimal Number of Components. 2. Age adjustments and distributional properties. 3. Inter-correlations among variables. 4. Non-independence among family members and ICA. 5. Table S1. Significant age covariates and percents of variance. 6. Table S2. Variable Characteristics. 7. Table S3. Correlation between individual variables. 8. Table S4. Kurtosis and Skewness of residuals. 9. Table S5. Correlation between ICA and family ID.

## Abbreviations

BP: Blood pressure
DBP: Diastolic blood pressure
GenSalt: Genetic Epidemiology Network of Salt Sensitivity
SBP: Systolic blood pressure
ICA: Independent component analysis
PCA: Principal components analysis
