# Supplementary material for: Aggregate blood pressure responses to serial dietary sodium and potassium intervention: defining responses using independent component analysis
Source: BMC Genet. 2015 Jun 20;16:64. doi: 10.1186/s12863-015-0226-8 (PMC4474450; doi:10.1186/s12863-015-0226-8)
Supplement: Additional file 1: — Additional material. 1. Optimal Number of Components. 2. Age adjustments and distributional properties. 3. Inter-correlations among variables. 4. Non-independence among family members and ICA. 5. Table S1. Significant age covariates and percents of variance. 6. Table S2. Variable Characteristics. 7. Table S3. Correlation between individual variables. 8. Table S4. Kurtosis and Skewness of residuals. 9. Table S5. Correlation between ICA and family ID. [file 12863_2015_226_MOESM1_ESM.doc]

**Additional material**

1. ***Optimal Number of Components***

Methods for determining the optimal number of components in PCA are subjective and often can yield an unrealistically large numbers of factors (27). Typical methods used to determine the optimal number of factors include (1) selecting all eigenvalues > 1 and (2) looking for points where the last big drop in eigenvalues occurs (i.e. slope levels off). Using the first criterion, the PCA-derived scree plot (see Figure 1) suggests 6 factors since there are 6 eigenvalues that are at least 1. However, the second method only picks up 4 factors before the slope levels off. Since both methods consistently suggest (at least) 4 factors, ICA results are presented for each of the 2-, 3- and 4-component solutions. Multiple (2-, 3- and 4-component) solutions were performed to examine the consistency and reliability of the factor structures.

1. ***Age adjustments and distributional properties***

First, sparse outliers (those beyond 4 standard deviations (SD) from the mean that are separated by at least 1 SD from the nearest internal point) are temporarily set aside so that extreme observations will not overly influence the age regression models. A given phenotype (P) is regressed on up to a 3rd-degree polynomial in age in a stepwise manner (P = f (age) + e). Only terms that are significant at the 5% level are retained. The residual variance from this regression (e2) also is examined for age effects (heteroscedasticity). The squared residual from the first regression is regressed on another polynomial in age in a stepwise manner (e^2^ = g (age) + w). Again, only terms that are significant at the 5% level are retained. To compute the final phenotype, the residual from the first (mean) regression is standardized by the square of the log transformed predicted score from the second regression. Although this regression model is developed after removing sparse outliers, all variables (including sparse outliers) are added back to the data for the computation of the final phenotype using model parameters developed earlier. These analyses are performed using the SAS/STAT software Version 9.3 for UNIX regression procedure. Age adjustment results are shown in Table 1s. Age effects in the mean are significant for most of the variables. However, heteroscedasticity (age effects in the variance) is found for only a few variables. Age effects account for between 0 and 19% of the variability in the mean and between 0 and 2.9% of the variability in the variance.

1. ***Inter-correlations among variables:***

The inter-correlations among variables are shown in table 3s. The expected pattern of higher inter-correlations among related variables (i.e. among BPs and among obesity) than across variable types is seen. Also, as expected, the correlations between the delta responses and the BPs during the various interventions are quite low. However, there is no strong evidence of any collinearity among the variables as the largest correlation is only 0.908.

1. ***Non-independence among Family Members and ICA***

While not much research has been done on how best to apply ICA to family-based samples, naïve or blind application of ICA to family-based samples can create some bias in the top components if there is confounding with family membership. One way to address this is to do stratified-sampling from each pedigree prior to application of the ICA. But, this is not practical here considering the loss of power due to small sample sizes. Another way is to check if there is a strong correlation between the ICAs and family ID. If there is a strong correlation then family ID should be adjusted out before testing for associations. However, as shown in table 5s, the correlations between family ID and the ICAs are very close to zero. Therefore, we are not overly concerned about the possible bias due to this approach.

**Table 1s.** Significant age covariates and percents of variance

| **Variable** | **Group** | **Mean Terms** | **R^2^ (%)** | | **Variance Terms** | **R^2^ (%)** |
| --- | --- | --- | --- | --- | --- | --- |
| B_SBP | Male | age | 2.38 | age^3^ | | 0.93 |
|  | Female | age | 12.17 | age | | 0.91 |
| B_DBP | Male | age, age^2^ | 7.37 | none | | -- |
|  | Female | age, age^3^ | 14.90 | none | | -- |
| L_SBP | Male | none | -- | none | | -- |
|  | Female | age | 6.86 | age | | 2.10 |
| L_DBP | Male | age, age^2^ | 8.30 | none | | -- |
|  | Female | age, age^3^ | 12.28 | none | | -- |
| H_SBP | Male | age | 1.67 | none | | -- |
|  | Female | age | 11.67 | age | | 1.84 |
| H_DBP | Male | age, age^2^, age^3^ | 10.05 | age^2^ | | 0.42 |
|  | Female | age, age^3^ | 14.18 | none | | -- |
| K_SBP | Male | none | -- | none | | -- |
|  | Female | age |  | age | | 2.10 |
| K_DBP | Male | age, age^2^, age^3^ | 11.20 | none | | -- |
|  | Female | age, age^3^ | 15.29 | none | | -- |
| B_Waist | Male | age, age^2^, age^3^ | 5.02 | none | |  |
|  | Female | age, age^2^ | 19.32 | age | | 0.54 |
| B_BMI | Male | age, age^2^, age^3^ | 4.03 | age | | 1.07 |
|  | Female | age, age^2^ | 11.94 | none | | -- |
| B_PP | Male | none | -- | none | | -- |
|  | Female | age^3^ | 2.94 | none | | -- |
| L_PP | Male | age, age^2^, age^3^ | 6.43 | none | | -- |
|  | Female | none | -- | none | | -- |
| H_PP | Male | age^2^, age^3^ | 3.90 | none | | -- |
|  | Female | age^2^, age^3^ | 3.35 | age^2^ | | 0.48 |
| K_PP | Male | age, age^3^ | 4.87 | none | | -- |
|  | Female | age^3^ | 0.47 | age^3^ | | 1.84 |
| D_LH_SBP | Male | age^2^ | 4.17 | none | | -- |
|  | Female | age^2^ | 6.12 | age | | 1.13 |
| D_LH_DBP | Male | age | 0.59 | | none | -- |
|  | Female | age^2^ | 1.13 | | age | 0.75 |
| D_HK_SBP | Male | age | 1.53 | | none | -- |
|  | Female | age^3^ | 2.46 | | age^3^ | 1.46 |
| D_HK_DBP | Male | none | -- | | none | -- |
|  | Female | none | -- | | none | -- |
| D_BL_SBP | Male | age^2^ | 5.05 | | age^3^ | 2.89 |
|  | Female | age | 6.94 | | age^3^ | 2.80 |
| D_BL_DBP | Male | none | -- | | none | -- |
|  | Female | age | 1.63 | | none | -- |

**Table 2s: Variable Characteristics**

| **Probands(N=640)** | | | | | | | | | | **Spouses(N=63)** | | | | | |  | | **Siblings(N=926)** | | | | | | | | | | **Offspring(N=192)** | | | | | | |  | | | |  | | | | |
| --- | --- | --- | --- | --- | --- | --- | --- | --- | --- | --- | --- | --- | --- | --- | --- | --- | --- | --- | --- | --- | --- | --- | --- | --- | --- | --- | --- | --- | --- | --- | --- | --- | --- | --- | --- | --- | --- | --- | --- | --- | --- | --- | --- |
| **Variable** | | | **Mean** | | **S** | | **SE** | | **Mean** | | | | **SE** | | | | **Mean** | | | |  | **SE** | | | | **Mean** | | | | | | **SE** | | | |  | | | |  | | | |
|  |  | | |  | | | |  | | | |  | | |  | | | | |  | | | | |  | | | | |  | | |  | | | | |  | | | |  |  |
| B_SBP | | 127.84 | | | | 0.45 | | | | | 111.96 | | | 1.82 | | | | | 111.54 | | | | 0.38 | | | |  | | 106.70 | | 0.75 | | | | | |  | | | |  | | |
| B_DBP | | 80.20 | | | | 0.35 | | | | | 72.19 | | | 1.21 | | | | | 71.05 | | | | 0.29 | | | |  | | 65.43 | | 0.65 | | | | | |  | | | |  | | |
| L_SBP | | 119.86 | | | | 0.44 | | | | | 106.23 | | | 1.48 | | | | | 107.19 | | | | 0.32 | | | |  | | 104.41 | | 0.71 | | | | | |  | | | |  | | |
| L_DBP | | 76.00 | | | | 0.36 | | | | | 69.33 | | | 1.09 | | | | | 69.09 | | | | 0.28 | | | |  | | 63.74 | | 0.64 | | | | | |  | | | |  | | |
| H_SBP | | 125.63 | | | | 0.49 | | | | | 112.04 | | | 1.70 | | | | | 111.82 | | | | 0.37 | | | |  | | 106.94 | | 0.73 | | | | | |  | | | |  | | |
| H_DBP | | 78.46 | | | | 0.37 | | | | | 71.37 | | | 1.20 | | | | | 70.81 | | | | 0.30 | | | |  | | 64.48 | | 0.63 | | | | | |  | | | |  | | |
| K_SBP | | 121.25 | | | | 0.49 | | | | | 107.87 | | | 1.61 | | | | | 108.63 | | | | 0.35 | | | |  | | 104.85 | | 0.74 | | | | | |  | | | |  | | |
| K_DBP | | 76.87 | | | | 0.36 | | | | | 69.44 | | | 1.20 | | | | | 69.50 | | | | 0.28 | | | |  | | 63.32 | | 0.64 | | | | | |  | | | |  | | |
| B_PP | | 47.65 | | | | 0.42 | | | | | 39.77 | | | 1.01 | | | | | 40.49 | | | | 0.24 | | | |  | | 41.27 | | 0.57 | | | | | |  | | | |  | | |
| L_PP | | 43.85 | | | | 0.43 | | | | | 36.90 | | | 0.94 | | | | | 38.11 | | | | 0.24 | | | |  | | 40.67 | | 0.59 | | | | | |  | | | |  | | |
| H_PP | | 47.17 | | | | 0.42 | | | | | 40.68 | | | 0.93 | | | | | 41.01 | | | | 0.24 | | | |  | | 42.46 | | 0.59 | | | | | |  | | | |  | | |
| K_PP | | 44.37 | | | | 0.44 | | | | | 38.44 | | | 0.88 | | | | | 39.13 | | | | 0.24 | | | |  | | 41.53 | | 0.61 | | | | | |  | | | |  | | |
| B2_WST | | 83.72 | | | | 0.38 | | | | | 80.50 | | | 1.25 | | | | | 79.46 | | | | 0.29 | | | |  | | 72.85 | | 0.74 | | | | | |  | | | |  | | |
| B_BMI | | 24.27 | | | | 0.13 | | | | | 23.40 | | | 0.46 | | | | | 23.08 | | | | 0.09 | | | |  | | 21.54 | | 0.24 | | | | | |  | | | |  | | |
| D_LH_SBP | | 5.77 | | | | 0.26 | | | | | 5.81 | | | 0.75 | | | | | 4.63 | | | | 0.19 | | | |  | | 2.53 | | 0.31 | | | | | |  | | | |  | | |
| D_LH_DBP | | 2.46 | | | | 0.23 | | | | | 2.03 | | | 0.55 | | | | | 1.73 | | | | | 0.17 | | |  | | 0.74 | | 0.36 | | |  | | | | | | |  | | |
| D_HK_SBP | | -4.38 | | | | 0.22 | | | | | -4.17 | | | 0.72 | | | | | -3.19 | | | | | 0.18 | | |  | | -2.09 | | 0.35 | | |  | | | | | | |  | | |
| D_HK_DBP | | -1.59 | | | | 0.18 | | | | | -1.93 | | | 0.51 | | | | | -1.31 | | | | | 0.15 | | |  | | -1.16 | | 0.33 | | |  | | | | | | |  | | |
| D_BL_SBP | | -7.99 | | | | 0.31 | | | | | -5.74 | | | 0.98 | | | | | -4.35 | | | | | 0.20 | | |  | | -2.30 | | 0.36 | | |  | | | | | | |  | | |
| D_BL_DBP | | -4.20 | | | | 0.23 | | | | | -2.86 | | | 0.64 | | | | | -1.97 | | | | | 0.17 | | |  | | -1.69 | | 0.39 | | |  | | | | | | |  | | |

**Table 3s: Correlation between individual variables**

|  | B_SBP | B_DBP | L_SBP | L_DBP | H_SBP | H_DBP | K_SBP | K_DBP | B2_WST | B_BMI |
| --- | --- | --- | --- | --- | --- | --- | --- | --- | --- | --- |
| B_SBP | 1 | 0.722 | 0.862 | 0.603 | 0.868 | 0.629 | 0.826 | 0.631 | 0.223 | 0.263 |
| B_DBP |  | 1 | 0.602 | 0.824 | 0.625 | 0.819 | 0.551 | 0.796 | 0.283 | 0.302 |
| L_SBP |  |  | 1 | 0.649 | 0.893 | 0.621 | 0.896 | 0.647 | 0.204 | 0.241 |
| L_DBP |  |  |  | 1 | 0.601 | 0.831 | 0.569 | 0.838 | 0.271 | 0.300 |
| H_SBP |  |  |  |  | 1 | 0.712 | 0.908 | 0.674 | 0.197 | 0.236 |
| H_DBP |  |  |  |  |  | 1 | 0.613 | 0.878 | 0.263 | 0.292 |
| K_SBP |  |  |  |  |  |  | 1 | 0.674 | 0.181 | 0.225 |
| K_DBP |  |  |  |  |  |  |  | 1 | 0.255 | 0.302 |
| B2_WST |  |  |  |  |  |  |  |  | 1 | 0.845 |
| B_BMI |  |  |  |  |  |  |  |  |  | 1 |

|  | B_SBP | B_DBP | L_SBP | L_DBP | H_SBP | H_DBP | K_SBP | K_DBP | B2_WST | B_BMI |
| --- | --- | --- | --- | --- | --- | --- | --- | --- | --- | --- |
| B_PP | 0.685 | 0.015 | 0.607 | 0.023 | 0.598 | 0.066 | 0.607 | 0.093 | 0.014 | 0.058 |
| L_PP | 0.52 | -0.032 | 0.645 | -0.143 | 0.558 | -0.008 | 0.588 | 0.018 | -0.007 | 0.015 |
| H_PP | 0.579 | 0.031 | 0.616 | -0.014 | 0.672 | -0.026 | 0.644 | 0.048 | 0.003 | 0.032 |
| K_PP | 0.506 | -0.031 | 0.580 | -0.050 | 0.573 | -0.023 | 0.683 | -0.047 | -0.006 | 0.013 |
| D_BL_SBP | -0.463 | -0.381 | 0.036 | -0.063 | -0.165 | -0.163 | -0.077 | -0.122 | -0.076 | -0.094 |
| D_BL_DBP | -0.273 | -0.396 | 0.008 | 0.193 | -0.107 | -0.073 | -0.033 | -0.021 | -0.048 | -0.034 |
| D_LH_SBP | 0.189 | 0.175 | -0.03 | 0.028 | 0.419 | 0.331 | 0.213 | 0.195 | 0.026 | 0.038 |
| D_LH_DBP | 0.122 | 0.084 | 0.037 | -0.191 | 0.275 | 0.381 | 0.155 | 0.157 | 0.015 | 0.016 |
| D_HK_SBP | -0.158 | -0.214 | -0.06 | -0.119 | -0.281 | -0.297 | 0.141 | -0.03 | -0.053 | -0.043 |
| D_HK_DBP | -0.066 | -0.135 | -0.021 | -0.074 | -0.158 | -0.341 | 0.067 | 0.147 | -0.043 | -0.010 |

|  | B_SBP | B_DBP | L_SBP | L_DBP | H_SBP | H_DBP | K_SBP | K_DBP | B2_WST | B_BMI |
| --- | --- | --- | --- | --- | --- | --- | --- | --- | --- | --- |
| B_PP | 0.685 | 0.015 | 0.607 | 0.023 | 0.598 | 0.066 | 0.607 | 0.093 | 0.014 | 0.058 |
| L_PP | 0.52 | -0.032 | 0.645 | -0.143 | 0.558 | -0.008 | 0.588 | 0.018 | -0.007 | 0.015 |
| H_PP | 0.579 | 0.031 | 0.616 | -0.014 | 0.672 | -0.026 | 0.644 | 0.048 | 0.003 | 0.032 |
| K_PP | 0.506 | -0.031 | 0.580 | -0.050 | 0.573 | -0.023 | 0.683 | -0.047 | -0.006 | 0.013 |
| D_BL_SBP | -0.463 | -0.381 | 0.036 | -0.063 | -0.165 | -0.163 | -0.077 | -0.122 | -0.076 | -0.094 |
| D_BL_DBP | -0.273 | -0.396 | 0.008 | 0.193 | -0.107 | -0.073 | -0.033 | -0.021 | -0.048 | -0.034 |
| D_LH_SBP | 0.189 | 0.175 | -0.03 | 0.028 | 0.419 | 0.331 | 0.213 | 0.195 | 0.026 | 0.038 |
| D_LH_DBP | 0.122 | 0.084 | 0.037 | -0.191 | 0.275 | 0.381 | 0.155 | 0.157 | 0.015 | 0.016 |
| D_HK_SBP | -0.158 | -0.214 | -0.06 | -0.119 | -0.281 | -0.297 | 0.141 | -0.03 | -0.053 | -0.043 |
| D_HK_DBP | -0.066 | -0.135 | -0.021 | -0.074 | -0.158 | -0.341 | 0.067 | 0.147 | -0.043 | -0.010 |

**Table 4s: Kurtosis and Skewness of residuals**

| Variable | Kurtosis | Skewness |
| --- | --- | --- |
| rB_SBP | -0.13 | 0.41 |
| rB_DBP | 0.09 | 0.30 |
| rL_SBP | 0.29 | 0.56 |
| rL_DBP | 0.42 | 0.38 |
| rH_SBP | 0.60 | 0.62 |
| rH_DBP | 0.39 | 0.34 |
| rK_SBP | 0.47 | 0.68 |
| rK_DBP | 0.36 | 0.43 |
| rB_PP | 0.54 | 0.74 |
| rL_PP | 0.71 | 0.85 |
| rH_PP | 0.70 | 0.81 |
| rK_PP | 0.81 | 0.89 |
| rB2_WST | 0.37 | 0.48 |
| rB_BMI | 0.73 | 0.69 |
| rD_LH_SBP | 1.29 | 0.53 |
| rD_LH_DBP | 0.65 | 0.15 |
| rD_HK_SBP | 2.92 | -0.43 |
| rD_HK_DBP | 1.44 | -0.15 |
| rD_BL_SBP | 2.03 | -0.73 |
| rD_BL_DBP | 1.14 | -0.09 |

**Table 5S: Correlation between ICA and family ID**

|  | ICA21 | ICA22 | ICA31 | ICA32 | ICA33 | ICA41 | ICA42 | ICA43 | ICA44 |
| --- | --- | --- | --- | --- | --- | --- | --- | --- | --- |
| Family ID | -0.01 | 0.129 | -0.025 | -0.111 | -0.189 | -0.089 | -0.049 | -0.099 | -0.203 |
